# Cystatin SA attenuates gastric cancer cells growth and increases sensitivity to oxaliplatin via PI3K/AKT signaling pathway

**DOI:** 10.1007/s00432-024-05780-9

**Published:** 2024-05-08

**Authors:** Yida Lu, Huizhen Wang, Sihan Chen, Bo Yang, Yaxian Li, Yongxiang Li

**Affiliations:** 1 Department of General Surgery, The First Affiliated Hospital of Anhui Medical University, Hefei, Anhui 230022 People’s Republic of China; 2 Taikang Ningbo Hospital, Ningbo, Zhejiang 315000 People’s Republic of China

**Keywords:** CST2, Gastric cancer, Oxaliplatin, PI3K/AKT

## Abstract

**Purpose:**

Cystatin SA (CST2) belongs to the superfamily of cysteine protease inhibitors. Emerging research indicates that CST2 is often dysregulated across various cancers. Its role and molecular mechanisms in gastric cancer remain underexplored. This study aims to explore the expression and function of CST2 in gastric cancer.

**Methods:**

CST2 expression was analyzed and validated through Western blot. CST2 overexpression was induced by lentivirus in GC cells, and the correlation between CST2 expression levels and downstream signaling pathways was assessed. In addition, multiple assays, including cell proliferation, colony formation, wound-healing, and transwell migration/invasion, were considered to ascertain the influence of CST2 overexpression on gastric cancer. The cell cycle and apoptosis were detected by flow cytometry.

**Results:**

CST2 expression at the protein level was decreased to be reduced in both gastric cancer tissues and cell lines, and CST2 expression attenuate gastric cancer growth, an effect restricted to gastric cancer cells and absent in gastric epithelial GES-1 cells. Furthermore, CST2 was demonstrated to improve chemosensitivity to Oxaliplatin in gastric cancer cells through the PI3K/AKT signaling pathway.

**Conclusion:**

These findings indicate that CST2 is downregulated at the protein level in gastric cancer tissues and cell lines. Additionally, CST2 was found to attenuate the growth of gastric cancer cells and to enhance sensitivity to Oxaliplatin through the PI3K/AKT signaling pathway, specific to gastric cancer cell lines. CST2 may serve as a tumor suppressor gene increasing sensitivity to Oxaliplatin in gastric cancer.

**Supplementary Information:**

The online version contains supplementary material available at 10.1007/s00432-024-05780-9.

## Introduction

Gastric cancer (GC) is one of the most aggressive and lethal malignancies of the digestive system, with a higher incidence in males than in females (Sung et al. 2021). The main risk factors include Helicobacter pylori infection, unhealthy diets, high alcohol consumption, and smoking (Campbell et al. 2007; Ishaq and Nunn 2015; Kim et al. 2014). The majority of GC cases are classified as adenocarcinomas, accounting for 80–90% (Casamayor et al. 2018). The WHO classifies GC into multiple subtypes, including papillary, tubular, mucinous, poorly cohesive carcinoma (signet-ring cell carcinoma and other variants), and mixed adenocarcinoma. The Lauren classification system consists of intestinal, diffuse, and indeterminate types (Bray et al. 2018).

Currently, treatment regimens against GC can be created from a molecular perspective, as many signaling pathways are abnormally regulated in GC cases (Clements et al. 2002; Feng et al. 2010). Although surgical resection is widely accepted as the main treatment for GC, chemotherapy remains a vital alternative, especially when resection is not feasible or the cancer is in metastatic stages. However, acquired resistance to chemotherapy agents limits their clinical efficacy in GC treatment. Nevertheless, the underlying molecular mechanisms of GC remain elusive. Therefore, further investigation of the unknown molecular mechanisms of GC is essential.

Cystatin SA (CST2) is a specific cysteine proteases inhibitor and a cystatin superfamily member. This superfamily is categorized into three main categories: type 1 (including CSTA, CSTB, etc.), which are predominantly intracellular proteins; type 2 (including CST1-7, etc.), which are primarily secretory proteins; and type 3, which are multi-domain proteins, such as mammalian kininogen (Koblinski et al. 2000; Lah et al. 1993). Several studies have found that members of the cystatin superfamily play important roles in inflammation and tumorigenesis. For instance, the upregulation of cystatin SN has been shown to promote hepatocellular carcinoma progression and is associated with a poor prognosis (Cui et al. 2019). Moreover, CST1 may serve as an independent prognostic factor for patients undergoing esophageal cancer surgery (Chen et al. 2013). CST6 exhibits upregulation in triple-negative breast cancer (Li et al. 2018). The expression level of CST2 is vital for the diagnosis of prostate cancer and may also influence drug efficacy in breast cancer. One study employed bioinformatics to identify CST2 as a potential prognostic marker in gastric cancer (Bao et al. 2019; Cheng et al. 2019; Liu et al. 2018). However, the specific roles and functions of CST2 in GC remain to be elucidated. Accordingly, in this study, we investigate CST2’s role in GC carcinogenesis and subsequently validated CST2’s functions using normal gastric cells, gastric cancer cells, and samples from GC patients. We ascertained that CST2 possesses the ability to suppress the malignant biological behaviors of gastric cancer cells, including cellular proliferation, migration, and invasion, and to enhance the chemosensitivity of gastric cancer cells to the chemotherapeutic agent oxaliplatin.

## Materials and methods

### GC sample collection

The samples of GC patients and their control samples were collected from surgical specimens from patients with GC between 2013 and 2017. All patients signed informed and received ethical approval to consent the use of human gastric tissue.

### GC cell lines culture

Human normal gastric epithelial cell line GES-1 and GC cell lines MGC803, SGC7901, HGC27, BGC823 and AGS were obtained from the Chinese Academy of Sciences (Shanghai, China). All cell lines were maintained in (RPMI)-1640 medium (R10-040-CV, Corning, USA), added with 10% FBS (FB25015, CLARK, USA) and 1% Penicillin–Streptomycin Solution (SV30010, HyClone, USA). All cell lines were maintained at 37 °C in an incubator containing a humidified 5% CO_2_ atmosphere.

### Cell transfection

We constructed a lentivirus overexpressing CST2 and a negative control lentivirus was purchased by Hanbio Technology Company (Shanghai, China). For lentiviral transfections, 5 × 10^4^ cells were pre-seeded in a 24-well plate overnight and transduced with the virus. After 48 h of transduction, cells were selected with 2 μg/ml puromycin (ST551, Sangon Biotech, China) for 6 days. The efficiency of overexpression was measured by western blot.

### Western blot (WB)

CST2 (Abcam, USA), GAPDH (BBI Life Sciences Corporation, China), PI3K and AKT antibodies (Santa Cruz Biotechnology, USA) were used to detect the expression of corresponding proteins by Western blot. Cell or tissue proteins were extracted using lysis buffer (78,501, Thermo Fisher Scientific, USA). Proteins was electrophoresed on Tris–Glycine SDS Running Buffer, and then transferred onto polyvinylidene fluoride membranes (ISEQ00010, Merck Millipore, Germany). The membranes were blocked with 5% non-fat milk (A600669, Sangon Biotech, China) and maintained overnight at 4 °C with specific antibodies. After incubation with secondary antibodies (1:5000, Thermo Fisher) for one hour at room temperature, The immune binding was detected using the ECL detection system (5200 multi, Tanon, China) (Sun et al. 2014).

### EdU staining

The BeyoClick™EdU Cell Proliferation Kit (C0078S, Beyotime, China) was used to assess cell proliferative ability. Approximately 5 × 10^4^ cells were seeded into 12-well cell culture plates and incubated overnight at 37 °C in an incubator. The cell were then trated with either DMSO or various reagents for 48 h. Subsequently, an equal volume of 20 μM EdU was added to the cell culture medium, and the cells were incubated for 120 min before being fixed with 4% PFA for 20 min. After fixation, the cells were then rinsed three times with 3% bovine serum albumin (BSA) and permeabilized for 15 min with in 0.3% Triton X-100 in PBS. Following permeabilization, the cells were incubated with BeyoClick™ Click Additive Solution at room temperature, protected from light for 30 min. Finally, the cell nuclei were stained with Hoechst 33,342 for 10 min at room temperature. The cells were imaged using a Live cell Imaging System (Cell discoverer 7, Carl Zeiss, Germany), and the number of cells that were positive for EdU in each field was calculated.

### Quantitative real-time PCR

Total RNA from both cells and tissues was extracted by using TRIzol reagent (Invitrogen, USA), and then used the ReverTra Ace qPCR RT Master Mix with gDNA Remover (Toyobo, Japan) according to the recommended protocol. Quantitative real-time PCR was conducted using KOD SYBR qPCR Mix (Toyobo, Japan). The primers used in this study were as follows CST2-F: GGAGGACAGGATAATCGAGGG, CST2-R: GTTCGGCCCACCTCTATGTC; GAPDH-F: CTCTGCTCCTCCTGTTCGAC, GAPDH-R: ACGACCAAATCCGTTGACTC.

### Cell counting Kit-8 (CCK-8) experiment

In total, 5 × 10^3^ cells were seeded on per well of a 96-well plate and cultured overnight for attachment. After a treatment with different concentrations of Oxaliplatin (MedChemExpress, USA) for 48 h, Cell counting Kit-8 (MedChemExpress, USA) was added to each well and cultured for 30, 60 and 90 min. Oxaliplatin crystals were dissolved in DMF before being added (the stock solution was 100 mM, and the working solution ranged from 0.1 to 10 mM). Then a Microplate reader (EXL800, BioTek Instruments, USA) was used to obtain the OD value of cells at 450 nm. The relative cell viability was calculated based on the absorbance of each well.

### Colony formation assay

In total, 500 cells were plated per well and maintained in a cell incubator for 12–14 days, with medium changes every 3 days. At the end of the experiment, cells were fixed with 4% formaldehyde for 20 min, stained with 0.1% crystal violet solution for another 20 min, followed by PBS washes several times. Colonies with more than 50 cells were counted. The efficiency of colony formation was calculated as [colonies counted/cells seeded × 100] %. All experiments were conducted in triplicate and repeated at least three times.

### Scratch wound assay

Transfected cells were seeded on 6-well plates one day prior and cultured until they reached 90% confluence. A linear wound in ever well was made in each well by scratching the confluent cell layer with 200 μL pipette tip. Cells were then rinsed three times with phosphate-buffered saline (PBS) to remove floating cells and debris. The wounds were photographed and measured at 0, 24 and 48 h using a Live Cell Imaging System (Cell discoverer 7, Carl Zeiss, Germany).

### Cell invasion assay

Cells were seeded on the upper chamber of a 24-well plate at a concentration of 5 × 10^5^ cells, which was coated with Matrigel (BD Biosciences, USA). RPMI-1640 containing 1–2% FBS was added to the upper chamber, while the lower chamber was filled with RPMI-1640 with 10% FBS. After 24–48 h, the cells that migrated through the upper chamber were fixed in 4% paraformaldehyde (PFA) and stained with 0.1% crystal violet. The stained cells were then photographed and counted under a microscope (DMi1, Leica, Germany).

### Immunofluorescence assay

Cells were seeded into a culture plate with pre-added cover glasses (Nest, China) and treated with various concentrations of Oxaliplatin. After 48 h, cells adhered to the cover glasses were fixed with 4% paraformaldehyde for 15 min at room temperature and washed three times with PBS. Cells were then permeabilized with 0.1% PBS-TX (0.1% Triton X-100 in PBS) for 5 min and washed three times with PBS. Cells were blocked in 2% BSA (bovine serum albumin), diluted in 0.1% PBS-TX, for 30 min, followed by the addition of 80 μL of 1X Phalloidin-iFluorTM 594 Conjugate (AAT Bioquest, USA) working solution, diluted 1:1000 in the blocking solution, for 1 h. Cells were then washed with PBS 3 to 5 times to remove excess solution. Subsequently, nuclei were stained with Antifade Mounting Medium with DAPI (P0131, Beyotime Institute of Biotechnology) for 15 min at room temperature. Confocal images were captured using a Live Cell Imaging System (Cell Discoverer 7, Carl Zeiss, Germany).

### Cell cycle distribution assay

The cell cycle distribution for each sample was detected by PI/RNase Staining Buffer Solution (550825, BD PharmingenTM, USA). After treatment with specific drugs, cells were trypsinized and collected in tubes, washed with PBS, and then fixed using 75% pre-cold ethanol at − 20 °C for at least overnight. Then, the cells were centrifuged at 12,000 rcf for 10 min, washed with PBS, and incubated in PI working solution for 20–30 min in the dark at room temperature. Cells were harvested using a flow cytometer at slow flow speed, and analyzed with ModFit LT cell cycle analysis software (Modfit LT 5.0; Verity Software House, Topsham, ME, USA).

### Nude mice xenograft experiments

4–6-week-old female nude mice (300–350 g) purchased from LINGCHANG BIOTECH (Shanghai, China) were randomly allocated to four groups: MGC803-CST2 group, MGC803-NC group, SGC7901-CST2 group and SGC7901-NC group. To establish the mouse xenograft model, nude mice were inoculated with 5 × 10^6^ GC cells dissolved in PBS into their right flank to induce tumors. Tumor volume was measured from the tenth day after injection and then measured every 3 days in three dimensions using a digital caliper. Tumor volume was calculated according to the formula: tumor volume (mm^3^) = π/6 × (W)^2^ × (L), where L represents the long diameter and W represents the short diameter. After 28 days, we used the IVIS Lumina XR Series III Imaging System (Perkin Elmer, USA) to obtain tumor fluorescence images before euthanizing the mice.. The tumors were then resected and measured.

### Ethics statement

All experiments involving animals follow the guidelines of the Animal Center of Anhui Medical University. Experimental protocols were approved by the Experimental Animal Ethical Committee of Anhui Medical University. All animals used in the study were euthanized at the end of this study.

### Statistical analysis

All experiments were repeated at least three times independently, and the data were assessed by GraphPad Prism 8 (version 8.0.1, GraphPad Software, La Jolla, CA, USA). Mean values are shown in the figures, and SDs are shown as error bars. Comparisons between treatments were assessed by a two-tailed Student’s *t*-test. All p values are labeled in the figures for where data were compared or between the experimental group and its control group.

## Results

### The expression of CST2 in gastric cancer and CST2 inhibits cell proliferation, migration and invasion in GC cells

**Fig. 1:**
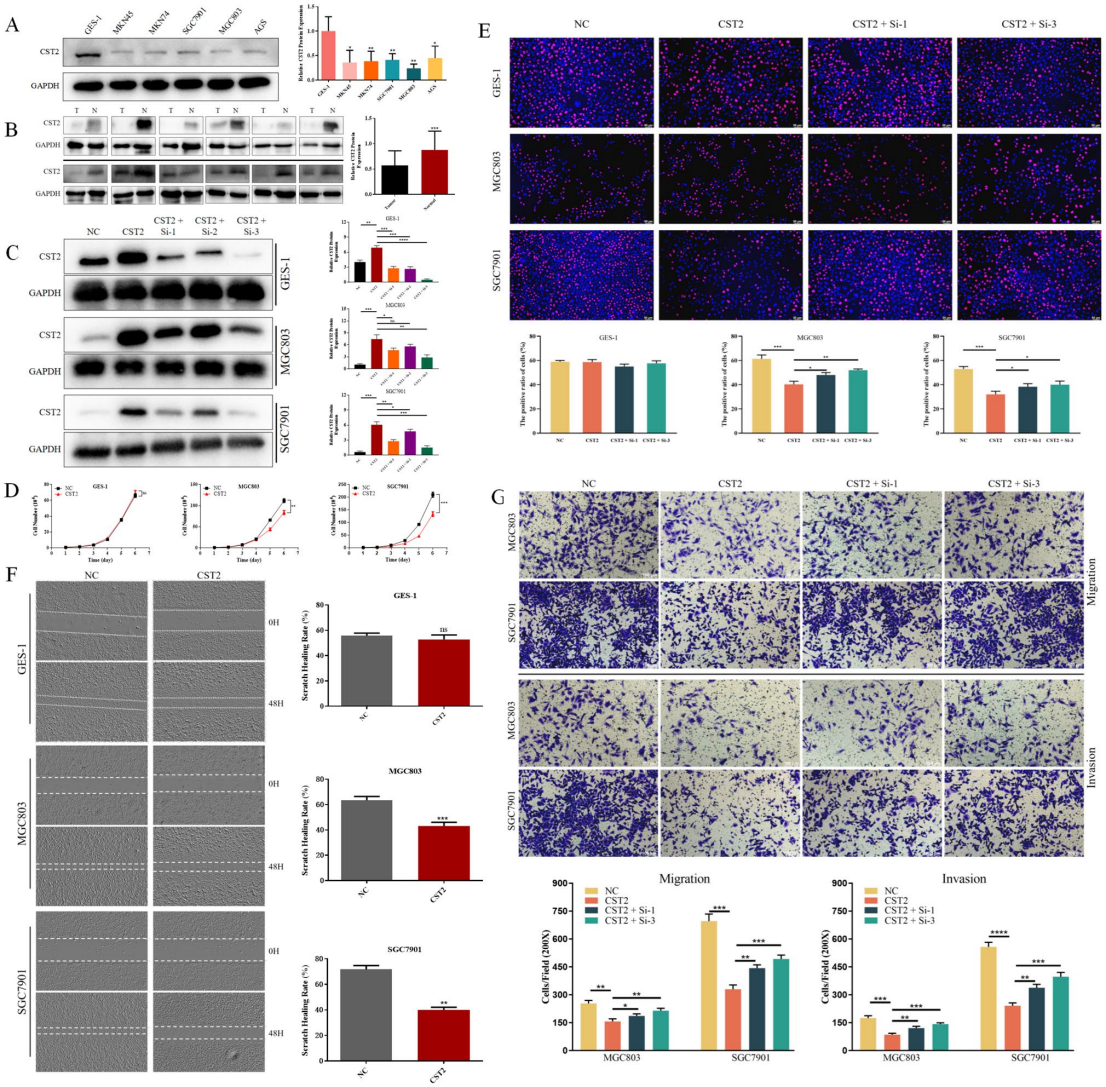
**A** Protein levels of CST2 in the normal gastric mucosal epithelial cell line GES-1 and various human GC cell lines. **B** The expression levels of CST2 protein in GC tissues compared to adjacent normal gastric tissues. **C** The overexpression and knockdown efficiency of CST2 were confirmed using Western blot analysis. **D** The proliferation curves were employed to compare proliferation rates between CST2 overexpression cells and control cells. **E** EdU staining was employed to assess the effect of CST2 overexpression and knockdown on GC cell proliferation. **F** Wound healing in cell monolayers was assessed at 48 h, and wound closure rates were calculated. (G) Cell migration and invasion abilities were evaluated using Transwell assays. Data are displayed as the mean ± SD (n = 3). *P < 0.05, **P < 0.01, ***P < 0.001

Three datasets gene expression profiles (GSE51575, GSE65801, and GSE79973) were retrieved from those present in the GEO database including both normal and gastric cancer samples. All differentially expressed genes (DEGs) were compared between normal controls and gastric cancer samples. These genes were further filtered and the Venn diagrams representing these genes were plotted (Fig. S1). CST2 was screened out to be further explored its effects on gastric cancer, according to our preliminary experiments. Western blot analysis was used to assess CST2 expression in cells and tissues. CST2 expression was lower in GC cell lines (MKN45, MKN74, SGC7901, MGC803, and AGS) compared to the normal gastric epithelial cell line GES-1 (Fig. 1A). Furthermore, CST2 protein expression was assessed in 12 pairs of fresh GC tissues and adjacent normal tissues, and a similar result was found, indicating that CST2 expression was decreased in GC tissues (Fig. 1B). The patients information is listed in Table 1.

**Table 1:** Patients information

Parameters	Gender	Age (years)	Tumor size (cm)	Tumor differentiation	Tumor location	Lymph nodes metastasis	TNM stage
Patient 1	Female	70	3.5 × 2 × 0.5	Poor	Antrum	Yes	IIB
Patient 2	Male	45	0.8 × 0.6 × 0.4	Poor	Antrum	Yes	IIB
Patient 3	Female	71	4 × 2.5 × 1.5	Well	Cardia	No	IIB
Patient 4	Female	52	6 × 4.8 × 1.8	Poor	Lesser curvature	Yes	IIIB
Patient 5	Female	50	3.5 × 3 × 0.7	Poor	Cardia	Yes	IIIA
Patient 6	Male	60	3.3 × 3 × 1.2	Moderate	Cardia	No	IB
Patient 7	Male	65	12 × 10 × 1.5	Poor	Body	Yes	IIIC
Patient 8	Male	73	4.0 × 2.5 × 1.0	Moderate	Cardia	Yes	IIIC
Patient 9	Male	57	4 × 3.5 × 1.8	Poor	Cardia	Yes	IIIB
Patient 10	Male	44	1.5 × 1.0 × 0.8	Moderate	Body	No	IA
Patient 11	Female	74	6.5 × 5 × 1.1	Moderate	Fundus	Yes	IIIC
Patient 12	Female	61	2 × 1 × 1	Moderate	Cardia	Yes	IIB

To further explore CST2’s role in GC carcinogenesis, GES-1, MGC803, and SGC7901 cells were selected for the overexpression and knockdown of CST2. The CST2 overexpression and knockdown efficiency were verified using Western blot (Fig. 1C).

Initially, 5000 GC cells were seeded per well in 12-well plates, and cell counting was conducted over the following 6 days. The proliferation curves, plotted based on the cell counting data, indicated that CST2-overexpression cells (MGC803-CST2/SGC7901-CST2) had lower proliferation rates compared to the negative control groups (MGC803/SGC7901-NC) (Fig. 1D). The number of MGC803 and SGC7901 cells decreased by 25.1% and 37.3%, respectively.

To validate the proliferation curves, EdU staining was performed on both CST2 overexpression and knockdown cells. The results also demonstrated that CST2 overexpression significantly inhibited GC cell proliferation (Fig. 1E), and CST2 knockdown could partially reverse the inhibitory effect. Consistently, both wound healing and cell invasion assays yielded similar results: Cells with CST2 overexpression exhibited reduced migration abilities compared to the control group, with scratch healing rates of MGC803 and SGC7901 cells decreasing by 20.4% and 31.8%, respectively (Fig. 1F). We then conducted transwell assays to assess cell invasion ability further, and the results similarly indicated that CST2 overexpression attenuated the invasive potential of GC cells. Notably, CST2 knockdown partially enhanced GC cells’ migration and invasion capacities (Fig. 1G). However, it was apparent that the inhibitory effect of CST2 on cell proliferation, migration, and invasion remained confined to the two GC cell lines and was not detected in the gastric epithelial cell line GES-1.

### CST2 suppresses tumor growth through the inhibition of the PI3K/AKT signaling pathway in mouse xenograft model

**Fig. 2:**
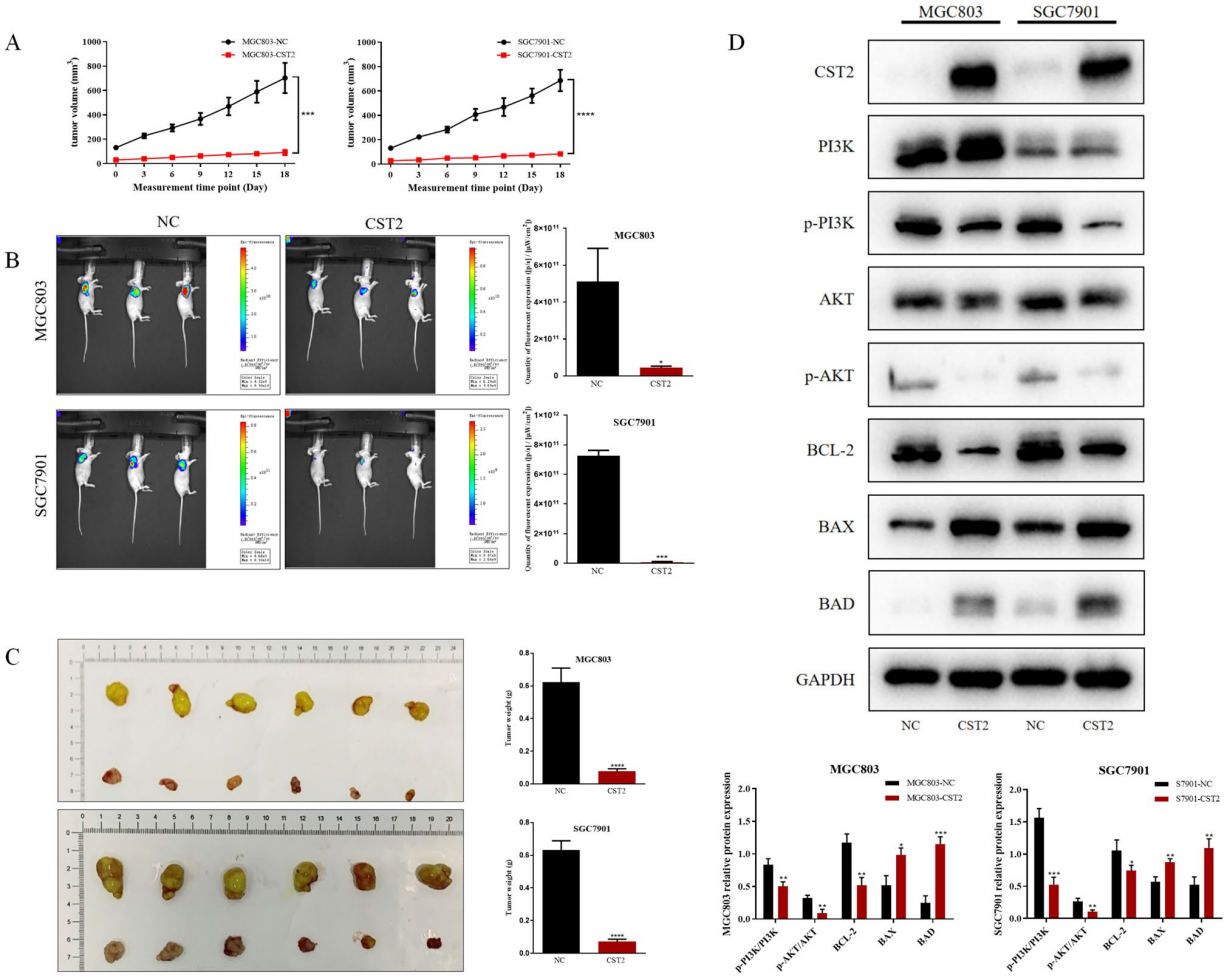
**A** Tumor volume for each group was recorded on the indicated days. **B** Images of the xenograft models were acquired using bioluminescence imaging, accompanied by a statistical analysis of tumor fluorescence intensity. **C** Tumors were dissected from mice post-euthanasia; subsequently, each tumor was collected and weighed. **D** Western blot analysis was performed to assess the expression of PI3K, p-PI3K, AKT, p-AKT, Bcl-2, BAX, and BAD in subcutaneous tumor samples of MGC803 and SGC7901 from each group. Data are displayed as the mean ± SD (n = 3). *P < 0.05, **P < 0.01, ***P < 0.001

To further examine CST2’s impact on GC tumor growth in vivo, CST2 overexpression cells (MGC803-CST2 and SGC7901-CST2), along with their corresponding control cells, were used to establish mouse xenograft models. Tumor volume measurements commenced on the 10th day post-injection and were conducted every 3 days after that (Fig. 2A). After 18 days of measurement, tumor fluorescence imaging was carried out prior to the euthanasia of the mice and tumor resection. Compared to the control group, slower tumor volume increases were noted in the MGC803-CST2 and SGC7901-CST2 groups. The fluorescent intensity readings ([p/s]/[μW/cm^2^]) provided consistent results. The average fluorescent intensity was reduced from 5.12 × 10^11^ to 4.53 × 10^10^ in the MGC803 group, and from 7.26 × 10^11^ to 7.63 × 10^9^ in the SGC7901 group (Fig. 2B). Additionally, the final tumor weights for the MGC803-NC and SGC7901-NC groups measured 0.632 g and 0.622 g, respectively, while the weights for the MGC803-CST2 and SGC7901-CST2 groups were substantially lower at 0.072 g and 0.077 g, representing reductions of 88.6% and 87.6%, respectively (Fig. 2C). To elucidate the underlying mechanisms attributed to CST2, protein expression levels within the PI3K/AKT signaling pathway and apoptosis were investigated. Western blot analysis showed that CST2 suppressed the expression of phospho-PI3K and phospho-AKT, two key components of the PI3K/AKT pathway. Furthermore, there was a significant elevation in the levels of pro-apoptotic proteins (BAX, BAD), while conversely, the level of the anti-apoptotic protein (BCL-2) was significantly reduced in the CST2 groups. Collectively, these data suggest that CST2 may suppress tumor growth by inhibiting the PI3K/AKT signaling pathway in vivo (Fig. 2D).

### SC79 reverses the effect of CST2 upregulation in gastric cancer cells

**Fig. 3:**
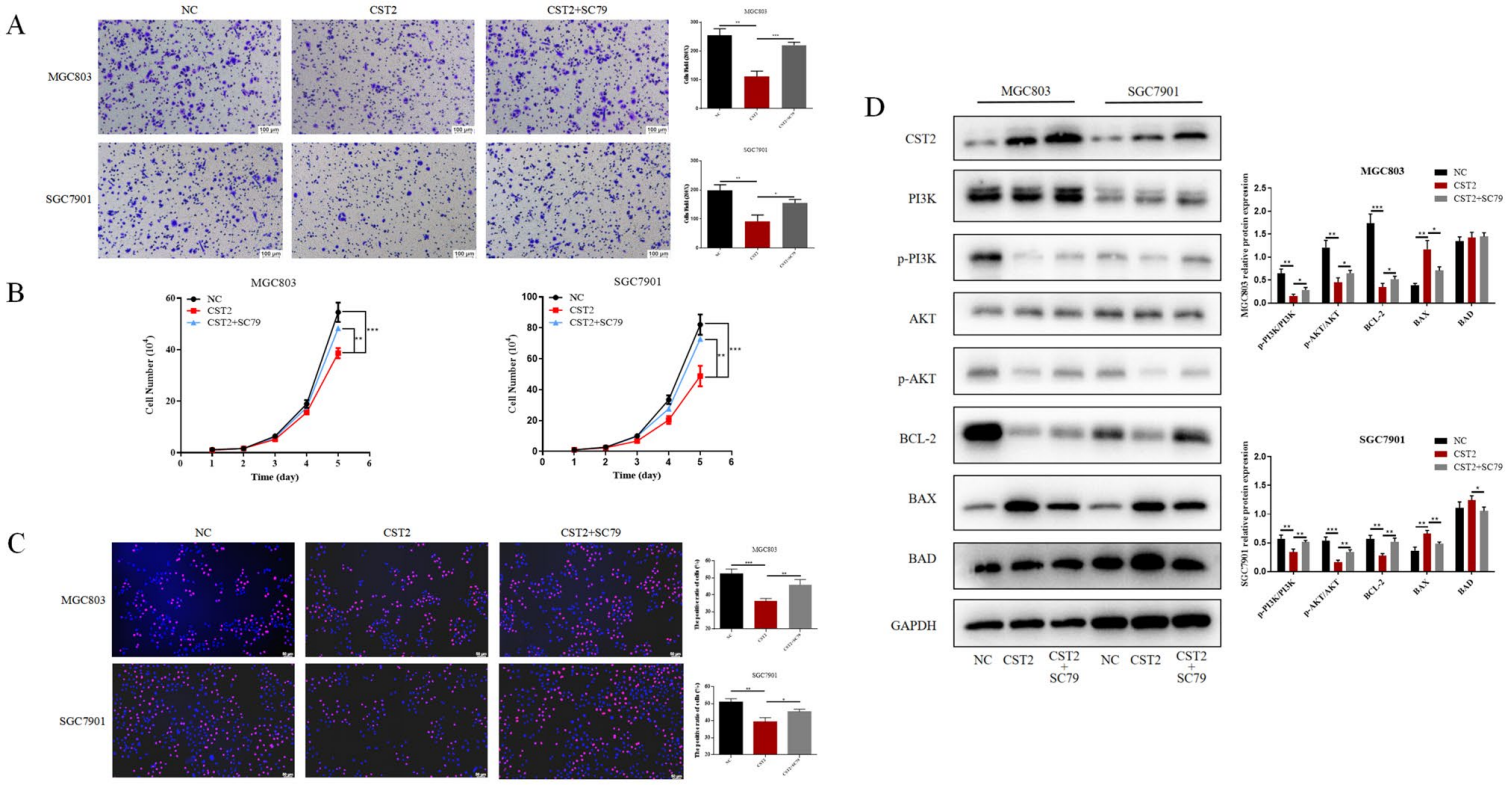
**A** Transwell assay to assess cell migration in NC (negative control), CST2-overexpressing, and CST2 + SC79-treated groups. **B**, **C** Cell proliferation in the two groups was assessed using cell proliferation curves and EdU staining. **D** Western blot analysis to assess the expression of PI3K, p-PI3K, AKT, p-AKT, Bcl-2, BAX, and BAD proteins in GC cells, with GAPDH serving as a loading control. Data are displayed as the mean ± SD (n = 3). *P < 0.05, **P < 0.01, ***P < 0.001

To further investigate whether the PI3K/AKT signaling pathway is vital for CST2-mediated antitumor effects, the AKT activator SC79 was administered to MGC803 and SGC7901 cell lines. The results showed that SC79 significantly counteracted the decreased cell migration ability observed in the CST2 overexpression cell lines (Fig. 3A). The proliferation curves and EdU staining results revealed that SC79 likewise counteracted the decreased cell proliferation associated with CST2 overexpression (Fig. 3B, C). Moreover, SC79 treatment increased the phosphorylation levels of PI3K and AKT and resulted in the restoration of the expression levels of Bcl-2, BAX, and BAD in cells overexpressing CST2 (Fig. 3D).

### CST2 upregulation contributes to oxaliplatin sensitivity via PI3K/AKT signaling pathway

Previous studies have shown that CST2 expression can potentially influence the efficacy of chemotherapy in cancer patients (Li et al. 2017; Qin et al. 2017). Given that oxaliplatin is a commonly used first-line chemotherapeutic agent for GC, we aimed to determine whether CST2 expression affects the therapeutic efficacy of oxaliplatin.

**Fig. 4:**
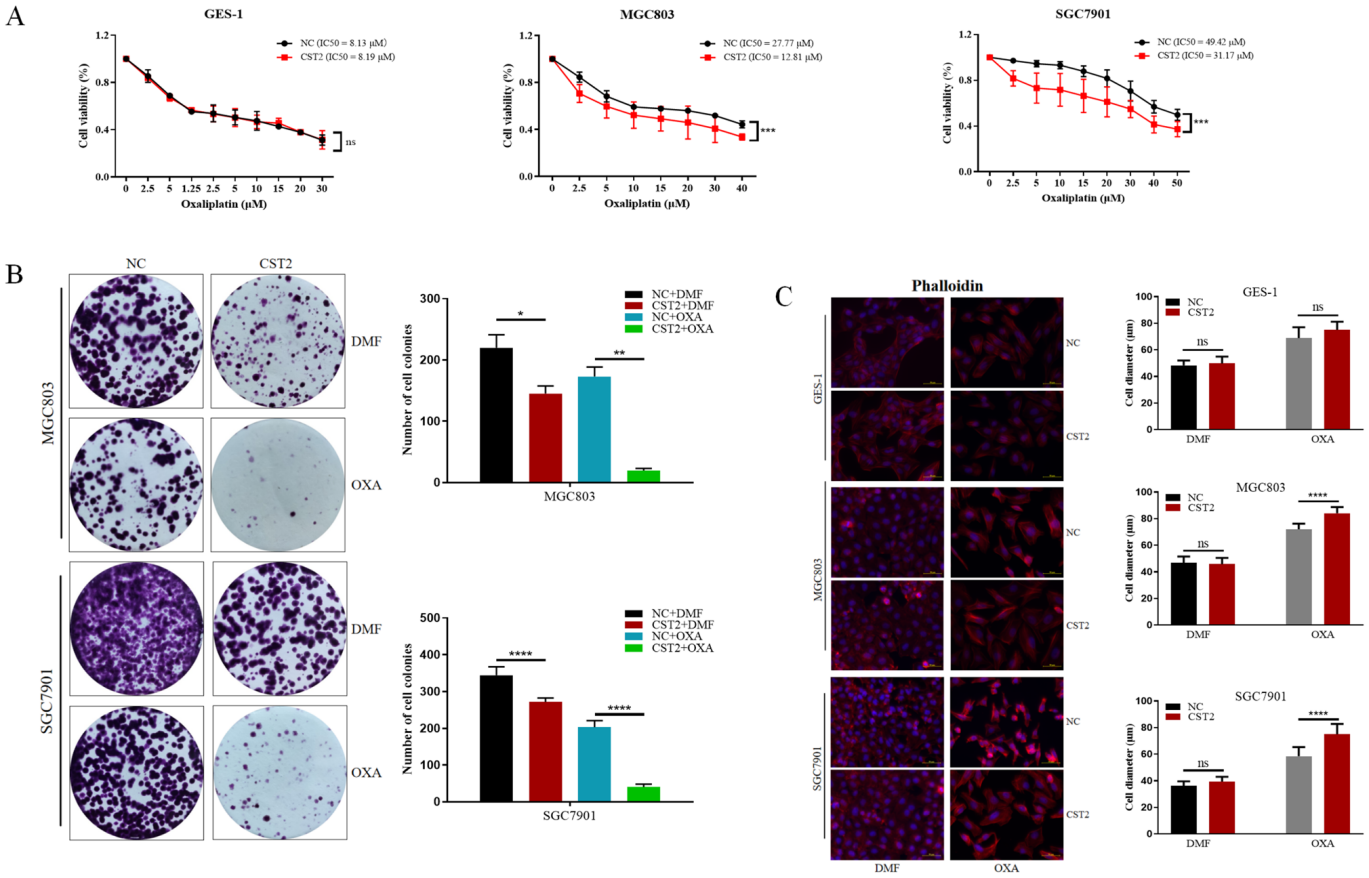
**A** The IC_50_ for oxaliplatin was determined for GES-1, MGC803, and SGC7901 cell lines using cell viability assays. **B** Cell colony formation assays were performed to confirm changes in oxaliplatin sensitivity among the three groups. The number of colonies was counted and graphically represented. **C** Following oxaliplatin treatment, differential immunofluorescence staining of phalloidin was used to visualize changes in microfilaments within GC cells. Data are displayed as the mean ± SD (n = 3). *P < 0.05, **P < 0.01, ***P < 0.001

A Cell Counting Kit-8 (CCK-8) assay was conducted to evaluate the cytotoxicity in CST2 overexpression and control cells, showing that CST2 upregulation could enhance chemosensitivity to oxaliplatin in GC cells. The IC_50_ of oxaliplatin for MGC803-CST2 cells (12.81 μM) proved to be substantially lower than that for the control cells (27.77 μM). In SGC7901 cells, the IC_50_ of oxaliplatin was reduced from 49.42 to 31.17 μM. However, such change was not observed in GES-1 cells (Fig. 4A).

Cell colony formation assays were conducted for MGC803/SGC7901-CST2 and control groups to validate these results further. We plated 500 cells in a 6-well plate and treated them with low doses of oxaliplatin (0.1 μM for MGC803 and 0.25 μM for SGC7901) for 12 days; the results showed that CST2 overexpression significantly inhibited colony formation in GC cells. GC cells also observed that treatment with 10 μg/mL oxaliplatin induced a change to a spindle-shaped morphology and cell scattering in GC cells (Fig. 4B).

Subsequently, an examination was undertaken to determine whether CST2 could regulate the reassembly of actin filaments in GES-1, MGC803, and SGC7901 cells. Fluorescent phalloidin staining was utilized to visualize the distribution of filamentous actin (F-actin). Oxaliplatin treatment was found to disrupt microfilament formation and to increase the area covered by microfilaments. Additionally, CST2 overexpression further disrupted microfilament formation and significantly enlarged the microfilaments. These findings indicate that CST2 overexpression may induce microfilament disruption following oxaliplatin treatment. However, these effects were not manifested in the gastric epithelial cell line GES-1 (Fig. 4C).

### CST2 facilitates cell cycle arrest and apoptosis induced by oxaliplatin in GC cells

**Fig. 5:**
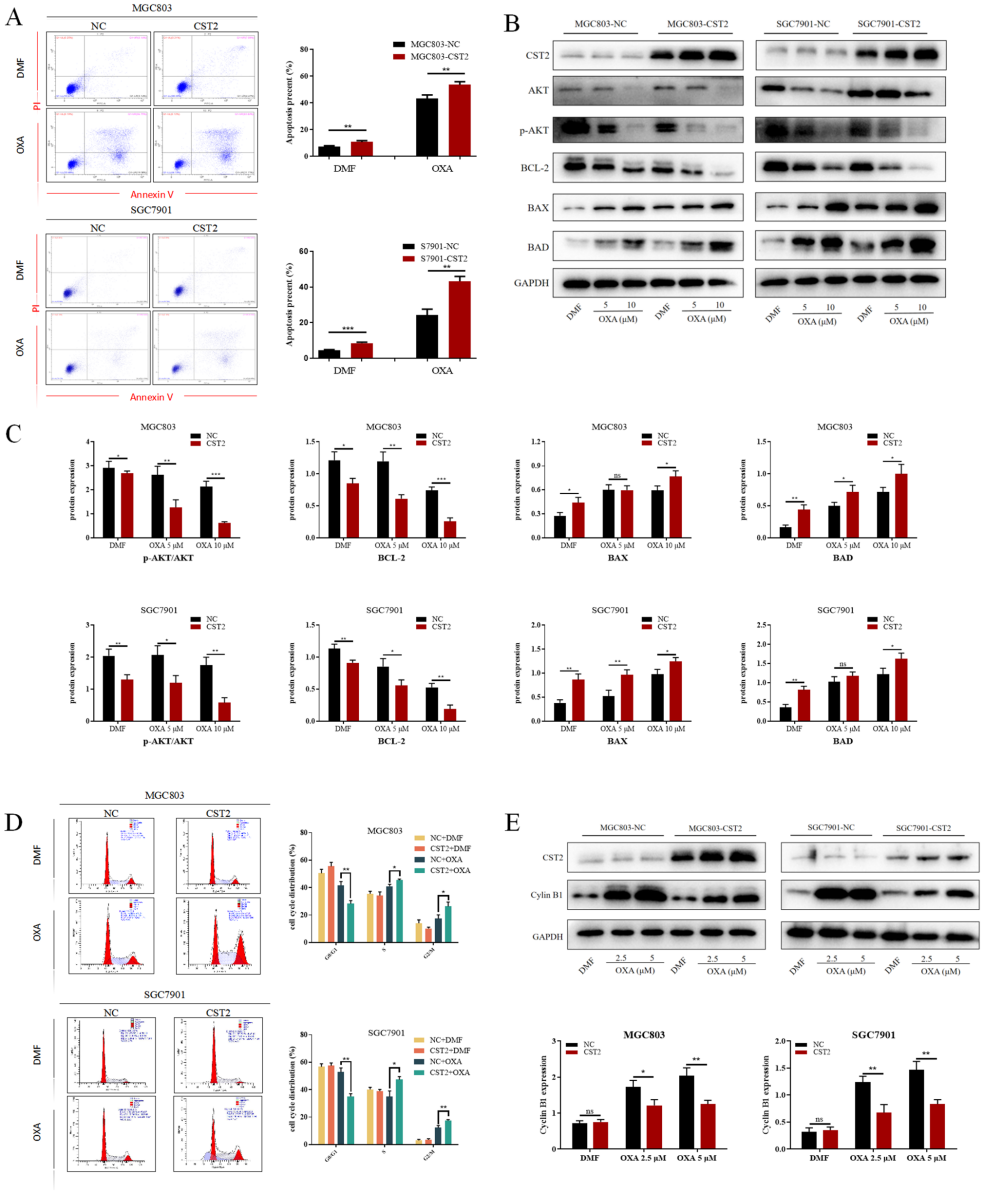
**A** The effects of CST2 on apoptosis of GC cells under oxaliplatin treatment were assessed by measuring the percentages of apoptotic cells in each group using flow cytometry. **B**, **C** Western blot analysis was carried out to measure the expression levels of AKT and apoptosis-related proteins in the representative groups. **D** Flow cytometry was used to analyze the effects of oxaliplatin on the cell cycle of various GC cell lines. **E** The expression levels of Cyclin B1 were assessed by Western blot analysis. Data are displayed as the mean ± SD (n = 3). *P < 0.05, **P < 0.01, ***P < 0.001

To explore the effects of CST2 on oxaliplatin response, MGC803 and SGC7901 cells were treated with low concentrations of oxaliplatin over 48 h. Flow cytometry analysis indicated that increased CST2 levels improved oxaliplatin-induced apoptosis in GC cells (Fig. 5A). Furthermore, CST2 overexpression showed favorable modulation in the protein levels of p-AKT, Bcl-2, BAX, and BAD, following oxaliplatin treatment (Fig. 5B, C). The impact of oxaliplatin on cell-cycle progression was also assessed using DNA content analysis via flow cytometry. Compared with control cells, cells overexpressing CST2 demonstrated a significant decrease in the percentage of G0/G1-phase cells and a lower proportion of cells in S and G2/M phases (Fig. 5D). Western blot analysis showed that the increased expression of Cyclin B1 protein following oxaliplatin treatment was significantly reduced in MGC803/SGC7901 cells with CST2 overexpression (Fig. 5E).

## Discussion

The development and progression of gastric cancer (GC) involves a complex interplay of numerous steps and genes. Therefore, understanding abnormal gene expression during carcinogenesis holds significant theoretical and practical importance. The high mortality rate associated with gastric cancer and its resistance to current chemotherapeutic agents highlight the urgent need for novel antitumor drugs, a primary focus for clinical researchers. Given the challenges in developing new antitumor agents, it is prudent to aim for reducing tumor resistance and enhancing the efficacy of chemotherapeutic agents (Lordick et al. 2022; Qu et al. 2023).

In our previous study, we confirmed and substantiated that CST2 RNA levels were inversely correlated with clinical data in the database. Unexpectedly, we discovered that CST2 was markedly expressed in tissues and exerted an anticancer effect (Xie et al. 2021). Furthermore, the literature lacks information on CST2 protein expression in GC tissues and cells. This observation could be attributed to the selective tumor inhibitory effect of CST2 on gastric cancer cells, sparing normal gastric epithelial cells. Investigating the potential relationship between these observations is crucial for guiding our subsequent research. If this relationship is linked to clinical drug resistance, it could improve the specificity of chemotherapeutic agents for gastric cancer cells and mitigate their cytotoxic effects on normal gastric epithelial cells, thereby enhancing chemotherapy outcomes for gastric cancer patients and reducing harm.

This study confirms that CST2 expression in gastric cancer tissues and cell lines is significantly lower than in normal gastric tissues and epithelial cells. Additionally, we generated multiple CST2 overexpression and knockdown cell models and validated the impact of CST2 expression levels on biological processes, including proliferation, apoptosis, migration, and invasion in GC cells. This was assessed through cell counting, colony formation, scratch wound healing, transwell migration/invasion assays, and flow cytometric analysis. These findings suggest that CST2 overexpression inhibits cell proliferation, migration, and invasion while enhancing the efficacy of oxaliplatin. Furthermore, we established a xenograft model in nude mice and observed that the CST2 overexpression significantly inhibited GC tumor growth in vivo compared to the control groups. Overall, our results indicate that CST2 overexpression has the potential to attenuate GC aggressiveness and progression both in vitro and in vivo.

The PI3K pathway is frequently dysregulated in human malignancies, regulating fundamental aspects of cancer such as cell proliferation, migration, metastasis, and survival (Alzahrani 2019; Gallardo et al. 2012). AKT, a serine-threonine kinase and a key target of PI3K, modulates numerous downstream target genes (Altomare and Testa 2005; Revathidevi and Munirajan 2019). To investigate the involvement of the PI3K/AKT signaling pathway in the oncogenic mechanism of CST2 in gastric cancer, we assessed the relationship between CST2 and p-PI3K, p-AKT, and other apoptosis-related proteins. Compared to the negative control (NC) groups, phosphorylation levels of PI3K and AKT were significantly reduced following CST2 overexpression, indicating CST2’s inhibition of PI3K/AKT pathway activation in gastric cancer. To further elucidate the correlation between CST2 overexpression, inhibition of gastric cancer cell proliferation and migration and decreased AKT phosphorylation, the AKT phosphorylation activator SC79 was utilized in vitro. The results suggest that AKT activation can counteract the inhibitory effects of CST2 overexpression on gastric cancer cells, implying that CST2 exerts its effects through the AKT pathway.

Commonly utilized drugs in gastric cancer chemotherapy induce adriamycin (ADR), platinum-based agents, 5-fluorouracil (5-FU), vincristine (VCR), and paclitaxel (PTX) (Rottenberg et al. 2021). Oxaliplatin, a second-generation platinum-based agent, is recommended for both adjuvant and palliative chemotherapy in gastric cancer treatment (Fong et al. 2022). Its principal cytotoxic mechanism involves the inhibition of DNA synthesis. Intrinsic or acquired resistance to oxaliplatin may lead to a poor prognosis. Oxaliplatin resistance in gastric cancer has been reported to be related to the PI3K/AKT pathway (Ren et al. 2023). Consequently, we explored the relationship between CST2, oxaliplatin sensitivity, and the PI3K/AKT pathway. The results demonstrated a significant enhancement of oxaliplatin sensitivity in gastric cancer cells overexpressing CST2. Upon exposure to equivalent concentrations of oxaliplatin, groups with CST2 overexpression showed significantly reduced levels of p-AKT and apoptosis-antagonizing proteins, as well as increased levels of pro-apoptotic proteins, compared to the control group. These findings suggest that CST2 may augment oxaliplatin sensitivity in gastric cancer cells by suppressing the PI3K/AKT pathway activity.

Previous studies have shown that the PI3K/AKT signaling pathway constitutes a pivotal nexus in cancer cells, governing cell growth, migration, proliferation, and metabolism. Targeting the oncogenic PI3K/AKT signaling pathway is currently considered a highly promising strategy for gastric cancer intervention. Apoptosis plays a crucial role in tumorigenesis, development, and drug resistance (Braicu et al. 2022; Yu et al. 2020). Suppression of the PI3K/AKT pathway induces cell apoptosis through diverse mechanisms, including modulating the activities of Bcl-2 family members and activating caspase family proteases (Kircher et al. 2019). In conclusion, our findings suggest that CST2 may modulate both the activity and the drug resistance of gastric cancer cells by inhibiting the PI3K/AKT signaling pathway. Furthermore, CST2 could suppress GC cell proliferation, migration, and invasion, while also augmenting the efficacy of oxaliplatin through the inhibition of the PI3K/AKT signaling pathway. This positions CST2 as a potential prognostic marker and therapeutic target for treating gastric cancer.

## Conclusions

We determined and confirmed that CST2 can suppress the malignant biological behaviors of gastric cancer cells, including cellular proliferation, migration, and invasion. Additionally, CST2 enhances the chemosensitivity of gastric cancer cells to the oxaliplatin, an effect observed specifically in gastric cancer cells without affecting normal gastric epithelial cells. We observed that the effects by CST2 on gastric cancer cells may be mediated by inhibition of PI3K/AKT pathway. To substantiate these obervations, we utilized the AKT activator SC79.

### Supplementary Information

Below is the link to the electronic supplementary material.

Supplementary file1 (TIF 2700 KB)

## Data Availability

The datasets used and/or analyzed in the current study are available from the corresponding author upon reasonable request.

## Abbreviations

CST2: Cystatin SA
GC: Gastric cancer
WB: Western blot
CCK-8: Cell counting Kit-8
