# Supplementary figures and images for: Cystatin SA attenuates gastric cancer cells growth and increases sensitivity to oxaliplatin via PI3K/AKT signaling pathway
Source: J Cancer Res Clin Oncol. 2024 May 8;150(5):244. doi: 10.1007/s00432-024-05780-9 (PMC11078793; doi:10.1007/s00432-024-05780-9)

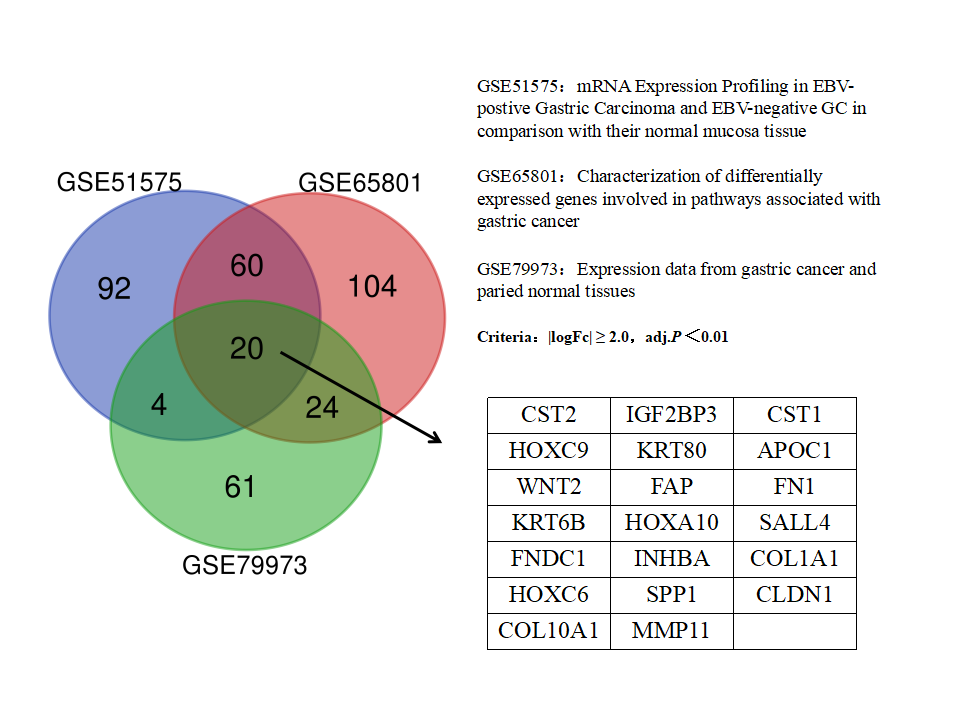

Supplement: Supplementary file 1 — Supplementary file1 (TIF 2700 KB) [file 432_2024_5780_MOESM1_ESM.tif]
